# The effect of interprofessional education on the work environment of health professionals: a scoping review

**DOI:** 10.1007/s10459-023-10300-4

**Published:** 2023-12-01

**Authors:** Mariana Medina-Córdoba, Sara Cadavid, Angela-Fernanda Espinosa-Aranzales, Karen Aguía-Rojas, Pablo Andrés Bermúdez-Hernández, Daniel-Alejandro Quiroga-Torres, William R. Rodríguez-Dueñas

**Affiliations:** 1 School of Medicine and Health Sciences, Universidad del Rosario, Bogota, Colombia; 2 Program of Psychology, People, Family and Society Research Group, School of Medicine and Health Sciences, Universidad del Rosario, Bogota, Colombia; 3 Program of Nursing, Public Health Research Group, School of Medicine and Health Sciences, Universidad del Rosario, Bogota, Colombia; 4 Program of Occupational Therapy, Rehabilitation Science Research Group, School of Medicine and Health Sciences, Universidad del Rosario, Bogota, Colombia; 5 Program of Medicine, Medical and Health Sciences Education Research Group, School of Medicine and Health Sciences, Universidad del Rosario, Bogota, Colombia; 6 Program of Biomedical Engineering, Gibiome Research Group, School of Medicine and Health Sciences, Universidad del Rosario, Bogota, Colombia; 7 Present Address: Electronics Engineering Department, Bioengineering Signal Analysis and Image Processing Research Group, Pontificia Universidad Javeriana, Bogota, Colombia

**Keywords:** Scoping review, Interprofessional education, Organizational environment, Health professionals, Interprofessional health care, Global health

## Abstract

To explore the existing literature on the effect of Interprofessional Education (IPE) on the work environment of health professionals. The research question was systematized according to the PCC (Population, Concept, and Context) format. A scoping review was performed. A search of multiple bibliographic databases identified 407 papers, of which 21 met the inclusion criteria. The populations of the 21 studies reviewed were composed of professionals in the fields of medicine, nursing, psychology, occupational therapy, physiotherapy, and social work, among others. The study contexts were both academic and nonacademic hospitals, mental health institutions, and community settings, and the topics examined were organizational climate, organizational culture, organizational attachment and job satisfaction. The findings from the reviewed studies showed positive effects of IPE interventions on organizational climate and culture, but the results on job satisfaction and organizational attachment were mixed (i.e., positive and no effects following IPE interventions). Research on IPE is worth more attention as IPE could be an effective alternative for the fulfillment of the Quadruple Aim and achieving the third of the United Nations Sustainable Development Goals, aimed at improving health and well-being. It seems critical for IPE to be positioned as a trend in global health, aiming at boosting human health resources as one of its building blocks and calling the attention of health decision-makers.

**Supplementary Information:**

The online version contains supplementary material available at 10.1007/s10459-023-10300-4.

## Introduction

The World Health Organization (WHO) defines health systems as the group of organizations, people, resources, and institutions seeking to improve the quality of health of consultants (World Health Organization, 2010). With the specific aim of improving health systems, the Triple Aim was proposed in 2008 (Interprofessional Educational Collaborative, 2016), whose three major objectives were: (1) improving the health of populations, (2) improving patients’ experience of care, and (3) reducing health care system costs. More recently, Brodenheimer and Sinsky (2014) updated this proposal and suggested the Quadruple Aim, adding the goal of (4) increasing health care professionals’ well-being and satisfaction within the experience of caring for others (Bodenheimer & Sinsky, 2014; Sikka et al., 2015). The Quadruple Aim approach considers that improving the well-being and satisfaction of the health care team should be as important as the three other objectives, given the rates of burnout and attrition of healthcare personnel, which directly impact the quality and costs of healthcare.

The WHO has also included the health human resource (HHR) as one of any health system’s components or building blocks. Indeed, the knowledge and skills with which HHR provides the service directly impact patients’ health (World Health Organization, 2010). In this regard, the HHR can be considered a crucial element for achieving the United Nations Sustainable Development Goals (SDGs). The SDGs comprehend 17 objectives proposed by the United Nations to improve the quality of life of people around the globe before 2030. These goals were thought to serve as a “shared blueprint for peace and prosperity for people and the planet, now and into the future” (Organización de las Naciones Unidas, 2018). The third SDG or SDG 3 is particularly relevant to this research: “ensure healthy lives and promote well-being for all at all ages” (Organización de las Naciones Unidas, 2018). The SDG 3 includes nine targets^1^ and four means of implementation of the targets, and one of these means (i.e., 3.c. SDG) refers specifically to the importance of the “recruitment, development, training and retention of the health workforce”.

Interprofessional Education (IPE) has been established as an essential strategy for achieving the goals of the Quadruple Aim and the third SDG (Interprofessional Educational Collaborative, 2016). On the one hand, as explained below, IPE strategies positively impact the HHR, which is precisely the Quadruple Aim’s fourth goal. On the other hand, as IPE also impacts health care quality, it can also contribute to the achievement of the SDG 3. The Quadruple Aim and the SDG 3 are part of a main framework connecting IPE to a Global Health approach.

The Center for the Advancement of Interprofessional Education (CAIPE, 2019) defines IPE as any activity in which “two or more professionals learn with, from, and about each other to improve collaboration in the healthcare field” (Buring et al., 2009), and the WHO (2010) observed that interprofessional training and education programs strengthen the collaborative work required to meet the new and coming global health challenges. Considering the abovementioned point, it is imperative to examine the potential effects of IPE initiatives on the six components of health systems as defined by WHO (World Health Organization, 2010): leadership and governance, service delivery, health system financing, health workforce, medical products, vaccines and technologies, and health information systems.

Interest in research on IPE has increased notably in recent years. A search conducted in Scopus in June 2023 of the term “interprofessional education” in title, abstract or keywords, showed that, between 1965 and 1999, only 63 articles were published, whereas between 2000 and 2009 there were 437 publications, and just between 2010 and June 22th 2023, a total of 5096 documents were published. This exponential growth coincides with the WHO’s (2010) publication of the “Framework for action for interprofessional education and collaborative practice” (World Health Organization, 2010). The significant rise in the number of publications on IPE makes it increasingly necessary to have rigorous literature reviews to obtain a clearer picture of the phenomena associated with the subject, and toward this end, researchers have conducted a growing number of systematic and scoping reviews. A search in Scopus of the terms “interprofessional education” AND “systematic review” in title, abstract, or keywords showed only 35 documents in the 15 years following the first records in 1999. Between 2014 and 2017, the search yielded another 35 documents, and between 2018 and 2023, we found 126 documents. The first scoping review found in Scopus with the terms “interprofessional education” AND “scoping review” in title, abstract, or keywords is from 2008. It was not until 2018 that the number of documents found per year was larger than two (five in 2018, six in 2019, and seven in 2020), and in recent years the numbers have scaled up to 13 in 2021, 24 in 2022, and another 10 articles so far in 2023.

The present study is a scoping review aimed at exploring whether IPE affects the work environment. A thorough exploration of previous systematic and scoping reviews showed us that, although researchers have addressed very important, diverse topics, none to the date have reviewed the effects of IPE on the work environment of health professionals. To our knowledge, no previous review has specifically explored whether IPE affects the work environment.

For example, in their review, Sirimsi et al. (2022) sought to identify strategies and interventions to improve interprofessional collaboration and integration (IPCI) in primary healthcare. The authors identified five overaching themes as generic strategies and methods that make IPCI easier in primary care. Hence, their focus was not the overall work environment, and their results were not framed within the work psychology conceptual field. Similarly, another recent scoping review on interprofessional education addressed interesting questions unrelated to the work environment framework, such as how IPE is implemented and the challenges faculty face when implementing IPE (Bogossian et al., 2023). Bogossian et al. (2023) specified whether the IPE strategies were applied in working (e.g., clinical) or classroom settings, but they focused on methodological characteristics and did not encompass the broad conceptual framework that we address in this review. Furthermore, our scoping review focuses specifically on whether IPE affects the work environment of health professionals in actual working settings.

Research that aims to explore the effect of IPE initiatives typically focuses on the effects observed on the service delivery and financing components of the health system. For example, Brashers et al. (2016) examined the effects of IPE initiatives on the clinical outcomes, quality, and costs of interventions. Other studies have emphasized the effect of IPE on patient satisfaction, adherence to treatment, user safety, and outcomes related to treatment efficacy and improvement (Moore et al., 2009; Walsh et al., 2014). A systematic review also revealed some positive outcomes associated with IPE, such as positive clinical outcomes for diabetes consultants, patient-centered communication, collaborative behavior, and reduced clinical errors (Reeves et al., 2013). However, the authors of that same systematic review remarked that there had been a paucity of rigorous and systematic research on the effects of IPE experiences on other components of health systems. Reeves et al. (2013) identified that more research is needed to understand the effects of IPE on (a) professional practice or health care outcomes, as well as on (b) relevant aspects of the work environment (e.g., communication among professionals, problems and complications resolution, work dynamics, and working conditions). Both the Quadruple Aim (Bodenheimer & Sinsky, 2014) and the SDGs (Organización de las Naciones Unidas, 2018) highlight the relevance of the well-being of the HHR in providing their professional services. In addition, according to one of the means of implementation of the SDG 3 (i.e., 3.c.), the HHR is a fundamental key to fulfilling the 2030 Agenda on Sustainable Development. The current paper focuses precisely on exploring the nature of the available literature on the effect of IPE among health professionals in their work settings.

The work environment is the group of physical and social environmental factors that influence the quality of life of the people who work in that specific environment, place or context. It includes both the areas in charge of preventing occupational diseases and those that focus on promoting the development and well-being of people while performing their professional activities (Knudsen et al., 2011). Within the work environment, four dimensions can be distinguished that are of interest for this research: organizational climate, organizational culture, organizational attachment (also called organizational commitment), and job satisfaction.

Regarding organizational climate, there is no consensus on its definition, so numerous definitions of this concept coexist. For this research, we used the definition of Méndez Álvarez (2006) because it is based on the fact that social interaction is what produces the organizational climate. Thus, like any human interaction, the organizational climate can be modified with an appropriate intervention such as IPE:

(…) “The company’s own environment, produced and perceived by each individual according to the conditions they find in their social interaction process and in the organizational structure (…) The social interaction process is the one where all the agents of change that can influence the shaping of organizational climate are generated” (…) (Méndez Álvarez, 2006, pp. 45–46).

Organizational culture is a dynamic phenomenon that is constantly shaped by social interactions within an institution, and is defined as the leadership, structures, rules, routines, and norms that direct the behavior of people within the institution (Schein, 2004). Although organizational climate and organizational culture are similar concepts, organizational culture can be considered more structural and is expressed in how the employees relate to each other within the institution.

We included two other constructs with significant interest for this review owing to their importance in the work environment and their possible interaction with interprofessional training applied in the workplace. The first phenomenon is organizational attachment, also called organizational commitment. This concept refers to the affective and behavioral state in which employees feel proud of and committed to the institution in which they work; they are involved and passionate regarding their roles within the organization (Macey & Schneider, 2008). The second concept is job satisfaction, which refers to employees’ states of positive affect toward their work (Macey & Schneider, 2008). Job satisfaction and organizational attachment appeared to correlate (Harter et al., 2002) and have been reported to be related to significant organizational outcomes. Within the work environment in which health professional teams are immersed, all the components described above play a key role.

Some dimensions of the work environment significantly affect clinicians’ communication and interaction (Lapo-Maza & Bustamante-Ubilla, 2018), which favors better decision-making in health care workers. Consequently, the quality of the health care professionals’ work environment has ethical implications in the care of consultants. In addition, the organizational environment of healthcare institutions plays a crucial role in professionals’ physical and mental well-being. Researchers have shown that a favorable work environment is related to patient satisfaction with care (Aoki & Guirardello, 2019), decreases in job abandonment (Copanitsanou et al., 2017), and reductions in therapeutic errors (van den Berg et al., 2017). Similarly, high job demands and low support are associated with increased stress-related disorders (Nieuwenhuijsen et al., 2010), and workplace bullying is related to increased absenteeism and turnover (Aoki & Guirardello, 2019). In addition, previous research has shown that the characteristics of the work environment significantly influence performance in clinical settings (Peña-Viveros et al., 2015).

Previous studies have found positive effects of IPE initiatives on the work environment of health care workers and the performance of work teams (Brashers et al., 2016) as well as on specific teamwork variables, such as increased mutual knowledge, contributions to collective work (Freeth & Nicol, 1998) and leadership related to team behavior, error correction in clinical scenarios and strengthened collective work (Morey et al., 2002). However, previous research does not always detail the professions of the participants who had engaged in IPE initiatives (i.e., several professions are labeled “other”), and does not specify the used IPE strategies (i.e., it does not detail whether they were debriefings, simulations…) (Brashers et al., 2016). Additionally, measurements on job satisfaction and involvement conducted with health professionals need to be expanded and systematized (Theobald et al., 2015), and there is a need to further explore the impacts of widescale IPE interventions given that current data only encompass local-level results (Reeves et al., 2017).

In short, the literature on the effects of IPE experiences on the work environment of the health care professionals is scant, and its results do not seem to be particularly conclusive. In addition, although there are reviews on the effect of IPE on collaborative practice and patient outcomes (Brashers et al., 2016; Reeves et al., 2013), we were unable to find any review on the effect of IPE on the work environment itself. In order to fill this gap in the literature, we have reviewed the literature on the effects of IPE on the work environment of health care professionals.

We chose a scoping review to explore the state of the current literature on the topic and to examine the similarities and differences between published papers (Tricco et al., 2018), including the variety of contexts and methodological strategies that have been applied to studying the relationship between IPE and work environment in clinical settings. Following our review, we aimed to identify the areas of work environment most impacted by IPE interventions and the specific IPE strategies that have been implemented to develop good collaborative practice at work. We expected to compile proposals that seek to increase the visibility, implementation, and efficiency of IPE as a strategy to improve health systems from a global point of view.

## Method

In the present scoping review, we aimed to review the state of the literature regarding the relationship between IPE programs and their effect on the work environment of health professionals. Furthermore, we aimed to analyze the state of the art on the subject and to review in depth the experiences of professionals participating in IPE programs. The review protocol was designed according to the type of study. We followed the recommended methodology and guidelines from the Joanna Briggs Institute (Peters et al., 2020) as well as the PRISMA reporting guidelines (Tricco et al., 2018) (see Supplementary Material 1). This scoping review included quantitative and qualitative studies for two reasons. First, we focused on accounting for the nature and variety of the existing literature, which is one of the goals of scoping reviews (Tricco et al., 2018), and this focus led us to obtain research based on both approaches and mixed approaches. Second, understanding the quantitative and subjective effects associated with IPE is important for health decision-makers to have a more complete view of the IPE experience when making decisions and generating health policies (Stern et al., 2020).

**Fig. 1:**
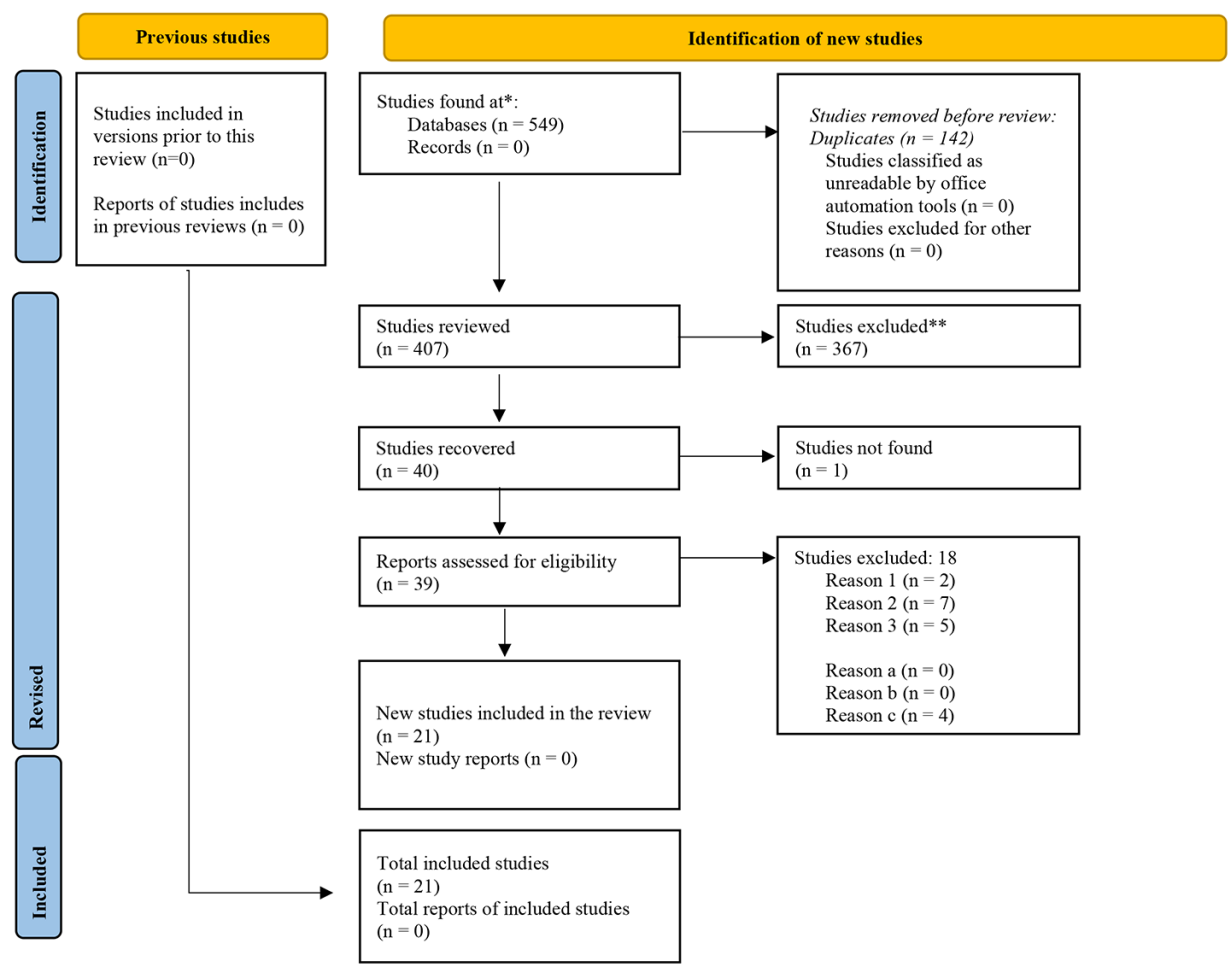
PRISMA flow diagram. *Note*: Reason 1 = the paper is a review. Reason 2 = the paper describes a protocol, without an intervention phase. Reason 3 = the paper evaluates a non-work environment, merely academic. Reason a = the paper defines an educational strategy that involves two or more professions (population). Reason b = the paper mentions a measure or a form of qualitative or quantitative evaluation of the effect of the educational strategy (concept) in the work environment in any of its dimensions: cooperation, communication, collaboration (context). Reason c = the paper is written in Spanish, English, French, or Portuguese

The research question was systematized according to the PCC format, in which the Population, Concept, and Context are described (Pollock et al., 2021). Table 1 contains the search strategy employed in each database. Each component of the research question is differentiated from the others by the Boolean marker AND, and terms corresponding to the same component were linked with the OR marker. In this review, the NOT marker was not considered necessary, and we did not impose a limit on years of publication because the intention was to review all the literature written to date on the effects of IPE on the work environment of health professionals. The following databases were used: Pubmed, Scopus, Scielo, Cochrane, PsycInfo, Eric, and Open Grey. The search strategy used in each case was tailored to the database characteristics and Scielo and Open Grey yielded no results. The calibration of each search strategy was performed and tested jointly by four authors, always based on the components of the question with PCC structure. The paper selection process is shown in Fig. 1.

**Table 1:** Search strategy for each database

Database	Search strategy	N° documents
Pubmed	(“Patient Care Team” [Mesh] OR “healthcare team” (subheading) OR “Health Personnel” [Mesh] OR “Students, Health Occupations” [Mesh] OR “health professionals”) AND (“Interprofessional education” OR “interprofessional training” OR “Interprofessional learning” OR “Simulation training” [MeSH] OR “Interprofessional education AND practice” OR “preceptorship”) AND (“Job Satisfaction” [MeSH] OR “Organizational Culture” [MeSH] OR “Work Engagement” [MeSH] OR “Work environment” OR “Organizational climate”).	284
Scopus	( TITLE-ABS-KEY (“Patient Care Team” OR “healthcare team” OR “Health Personnel” OR “Students,Health Occupations” OR “health professionals”) AND TITLE-ABS-KEY (“Interprofessional education” OR “interprofessional training” OR “Interprofessional learning” OR “Simulation training” OR “Interprofessional education AND practice” OR “preceptorship”) AND TITLE-ABS-KEY (“Job Satisfaction” OR “Organizational Culture” OR “Work Engagement” OR “Work environment” OR “Organizational climate”) )	226
Scielo	(“Patient Care Team” OR “healthcare team” (subheading) OR “Health Personnel” OR “Students, Health Occupations” OR “health professionals”) AND (“Interprofessional education” OR “interprofessional training” OR “Interprofessional learning” OR “Simulation training” OR “Interprofessional education AND practice” OR “preceptorship”) AND (“Job Satisfaction” OR “Organizational Culture” OR “Work Engagement” OR “Work environment” OR “Organizational climate”)	0
Cochrane	(((((Patient Care Team or healthcare team or Health Personnel or Students Health Occupations or health professionals) and Interprofessional education) or interprofessional training or Interprofessional learning or Simulation training or (Interprofessional education and practice) or Preceptorship) and Job Satisfaction or Organizational Culture or Work Engagement or Work environment or Organizational climate) limit 1 to (journal article or clinical trial or “review” or clinical trial,all or controlled clinical trial) [Limit not valid in CDSR,ACP Journal Club,DARE,CCA,CCTR,CLCMR,CLHTA,CLEED; records were retained]	16
PsycInfo	(“Patient Care Team” OR “health professionals” OR “healthcare team” OR “Health Personnel” OR “Students, Health Occupations” OR ) AND (“Interprofessional education” OR “Interprofessional learning” OR “interprofessional training” OR “Simulation training” OR “Interprofessional education” OR “preceptorship”) AND (“Job Satisfaction” OR “organizational culture” OR “work engagement” OR “work environment” OR “organizational climate”)	11
ERIC	(“Patient Care Team” OR “health professionals” OR “healthcare team” OR “Health Personnel” OR “Students, Health Occupations”) AND (“Interprofessional education” OR “Interprofessional learning” OR “interprofessional training” OR “Simulation training” OR “Interprofessional education” OR “preceptorship”) AND (“Job Satisfaction” OR “organizational culture” OR “work engagement” OR “work environment” OR “organizational climate” OR “professional identity”)	12
Open Grey	(“Patient Care Team” OR “health professionals” OR “healthcare team” OR “Health Personnel” OR “Students, Health Occupations”) AND (“Interprofessional education” OR “Interprofessional learning” OR “interprofessional training” OR “Simulation training” OR “Interprofessional education” OR “preceptorship”) AND (“Job Satisfaction” OR “organizational culture” OR “work engagement” OR “work environment” OR “organizational climate” OR “professional identity”)	0

We selected papers based on the eligibility criteria we list below were established for the scientific papers to be reviewed on the selected subject:

**Inclusion criteria**

- The paper defines an educational strategy involving two or more professions (population).
- The paper mentions a qualitative or quantitative measurement of the effect of the educational strategy on the work environment in any of its dimensions: climate, culture, job satisfaction, and organizational attachment/organizational commitment (concept).
- The text of the paper is written in English, Spanish, French or Portuguese.

**Exclusion criteria**

1. The paper is a review.
2. The paper describes protocols for implementing educational strategies without reporting results.
3. The paper evaluates the effect in an academic or other non-work environment.

For the abstract review and the paper selection process, we used the Rayyan office tool (Ouzzani et al., 2016) to upload the lists of papers we found in each database. Once uploaded, duplicated papers were identified and excluded from the initial sample of abstracts. Then, five of the six authors performed an inclusion/exclusion criteria calibration exercise (Peters et al., 2020) with 25 papers. If 70% agreement was not achieved, a third investigator reviewed the abstract and determined its inclusion or exclusion following a discussion. We tracked this process on a spreadsheet designed for this purpose.

Since there was no 70% agreement, a second calibration exercise was performed with another 25 papers, in which a 68% agreement was achieved. Following a new discussion among the authors, consensus was reached, so selection standardization was completed. Following the calibration, pairs of researchers independently read the abstracts of the papers to assess their suitability based on the inclusion and exclusion criteria. When a discrepancy arose within a pair, they discussed it so a consensus was reached. In cases where the pair did not reach an agreement, all the review team discussed the eligibility of the conflicting paper.

Subsequently, all the authors constructed a table for recording and extracting data from the studies. The table was tested with one of the papers to evaluate the level of agreement on the data to be extracted. This process was repeated twice until all doubts about the extraction process were clarified. The data recording and extraction table was constructed according to the study’s objectives and the PCC structure of the research question. Thus, the authors determined categories for extracting the country of the study, type of study, and all the categories necessary to describe the population (sociodemographic characteristics and professions), the context (clinical or community), and the concept. Within this last category, we included the IPE strategies (their name, type, application, implementation time, data collection modality, and evaluation) and the work environment dimensions of interest (climate, culture, attachment/commitment, and satisfaction).

Since this was a scoping review, the study population was not considered to be at risk (i.e., there was no direct contact with the participants). All the ethical principles of the research were preserved, such as care in preserving the data quality and methodological rigor.

## Results

From our review, we identified 407 deduplicated papers. The 407 abstracts were then reviewed to control for selection bias. Considering the inclusion and exclusion criteria described above, 39 abstracts were included and 367 were excluded. Only one paper was found without an abstract and we consulted the bibliographic catalogs of six institutions to find it, with no results: Universidad de los Andes, Universidad del Rosario, Universidad Nacional de Colombia, University of Glasgow, Universidad de Salamanca, and Duke University. After the full-text reading of the 39 identified papers, we excluded 18 papers that did not meet the abovementioned inclusion and exclusion criteria. Thus, 21 documents met the established criteria and were included in the current review (Fig. 1). The most relevant characteristics of each paper can be found in Supplementary Material 2.

Significant methodological variability was observed in the papers. Specifically, five documents were found to have a qualitative approach (Brewer & Flavell, 2020; Christofilos et al., 2015; Dematteo & Reeves, 2011; Jowsey et al., 2019; Rider et al., 2019), among which there were two exploratory phenomenological papers (Brewer & Flavell, 2020; Jowsey et al., 2019), two based on grounded theory (Christofilos et al., 2015; Rider et al., 2019) and one case study (Dematteo & Reeves, 2011). In addition, there were six mixed-methods studies (Bajnok et al., 2012; Carney et al., 2019; Kolbe et al., 2013; Lee et al., 2021; Nagelkerk et al., 2014; Slater et al., 2012) and ten quantitative studies (Braithwaite et al., 2013; Gros et al., 2021; Hinde et al., 2016; Marrone, 2018; Meurling et al., 2013; Roberts et al., 2014; Tahtali et al., 2017; Villemure et al., 2019; Wong et al., 2016). Among these, six papers were exploratory (Braithwaite et al., 2013; Gros et al., 2021; Marrone, 2018; Meurling et al., 2013; Pullon & Fry, 2005; Wong et al., 2016), two were longitudinal (Hinde et al., 2016; Roberts et al., 2014), and two were quasi-experimental (Tahtali et al., 2017; Villemure et al., 2019).

The relevant results from each source of evidence were arranged according to the PCC (i.e., population, concept, and context) question format (see Supplementary Material 2). With respect to context, we will now address the countries where the interventions were carried out and the specific settings in which the IPE strategies were implemented. Regarding the countries, we found that all studies were conducted in countries belonging to the Global North, such as the United States (Carney et al., 2019; Dematteo & Reeves, 2011; Gros et al., 2021; Lee et al., 2021; Marrone, 2018; Nagelkerk et al., 2014; Rider et al., 2019; Roberts et al., 2014; Wong et al., 2016), Canada (Bajnok et al., 2012; Christofilos et al., 2015; Villemure et al., 2019), Australia (Braithwaite et al., 2013; Brewer & Flavell, 2020), New Zealand (Jowsey et al., 2019; Pullon & Fry, 2005), United Kingdom (Hinde et al., 2016; Slater et al., 2012), Germany (Tahtali et al., 2017), Sweden (Meurling et al., 2013) and Switzerland (Kolbe et al., 2013). The clinical settings were heterogeneous, with a predominance of university/teaching hospitals (Christofilos et al., 2015; Dematteo & Reeves, 2011; Gros et al., 2021; Kolbe et al., 2013; Marrone, 2018; Meurling et al., 2013; Roberts et al., 2014; Villemure et al., 2019; Wong et al., 2016). We also found non-teaching hospitals (Bajnok et al., 2012; Carney et al., 2019; Hinde et al., 2016; Lee et al., 2021; Nagelkerk et al., 2014; Pullon & Fry, 2005; Slater et al., 2012), primary care residencysetting (Carney et al., 2019), district health boards (Jowsey et al., 2019), community care centers (Bajnok et al., 2012; Brewer & Flavell, 2020), rehabilitation and health science centers (Bajnok et al., 2012), mental health hospitals and individual general practices (Slater et al., 2012), interprofessional training organizations (Rider et al., 2019), and networks of health services (Braithwaite et al., 2013).

Concerning the population examined in he literature, we found that most of the studies included physicians (Bajnok et al., 2012; Braithwaite et al., 2013; Carney et al., 2019; Christofilos et al., 2015; Dematteo & Reeves, 2011; Gros et al., 2021; Hinde et al., 2016; Jowsey et al., 2019; Kolbe et al., 2013; Lee et al., 2021; Marrone, 2018; Meurling et al., 2013; Nagelkerk et al., 2014; Pullon & Fry, 2005; Rider et al., 2019; Roberts et al., 2014; Slater et al., 2012; Tahtali et al., 2017; Villemure et al., 2019; Wong et al., 2016) and nursing professionals (Bajnok et al., 2012; Braithwaite et al., 2013; Brewer & Flavell, 2020; Carney et al., 2019; Christofilos et al., 2015; Dematteo & Reeves, 2011; Gros et al., 2021; Hinde et al., 2016; Jowsey et al., 2019; Kolbe et al., 2013; Lee et al., 2021; Marrone, 2018; Meurling et al., 2013; Nagelkerk et al., 2014; Pullon & Fry, 2005; Rider et al., 2019; Roberts et al., 2014; Slater et al., 2012; Tahtali et al., 2017; Villemure et al., 2019; Wong et al., 2016). Several studies included professionals from other areas, such as physiotherapists (Bajnok et al., 2012; Brewer & Flavell, 2020; Christofilos et al., 2015; Dematteo & Reeves, 2011), psychologists (Brewer & Flavell, 2020; Carney et al., 2019; Dematteo & Reeves, 2011; Rider et al., 2019), occupational therapists (Bajnok et al., 2012; Brewer & Flavell, 2020; Dematteo & Reeves, 2011; Slater et al., 2012), nutritionists (Bajnok et al., 2012), speech therapists (Bajnok et al., 2012; Brewer & Flavell, 2020; Dematteo & Reeves, 2011); pharmacists (Carney et al., 2019; Dematteo & Reeves, 2011; Slater et al., 2012), social workers (Bajnok et al., 2012; Christofilos et al., 2015; Dematteo & Reeves, 2011; Rider et al., 2019; Slater et al., 2012), respiratory therapists (Roberts et al., 2014; Villemure et al., 2019), recreational therapists (Bajnok et al., 2012), clinical management professionals (Dematteo & Reeves, 2011; Pullon & Fry, 2005), trainers (Carney et al., 2019), guidance counselors (Dematteo & Reeves, 2011), administrative staff (Bajnok et al., 2012; Braithwaite et al., 2013; Carney et al., 2019; Jowsey et al., 2019; Rider et al., 2019; Slater et al., 2012), supervisors (Carney et al., 2019; Roberts et al., 2014) and clinical researchers (Rider et al., 2019). Additionally, some studies counted on the participation of technical (Bajnok et al., 2012; Gros et al., 2021; Lee et al., 2021; Nagelkerk et al., 2014; Pullon & Fry, 2005; Rider et al., 2019; Roberts et al., 2014; Tahtali et al., 2017), and support (Bajnok et al., 2012; Carney et al., 2019; Gros et al., 2021; Hinde et al., 2016; Jowsey et al., 2019; Marrone, 2018; Meurling et al., 2013; Villemure et al., 2019) personnel.

Regarding our concept, our research focus was interprofessional education and training interventions and their effects on the work environments of health professionals. We found a predominance of simulation strategies (Carney et al., 2019; Dematteo & Reeves, 2011; Gros et al., 2021; Hinde et al., 2016; Jowsey et al., 2019; Kolbe et al., 2013; Lee et al., 2021; Meurling et al., 2013; Nagelkerk et al., 2014; Pullon & Fry, 2005; Roberts et al., 2014; Tahtali et al., 2017; Villemure et al., 2019; Wong et al., 2016), nine of which were accompanied by debriefing (Dematteo & Reeves, 2011; Gros et al., 2021; Jowsey et al., 2019; Kolbe et al., 2013; Lee et al., 2021; Meurling et al., 2013; Nagelkerk et al., 2014; Roberts et al., 2014; Villemure et al., 2019), one of them with workshop sessions and theoretical training (Pullon & Fry, 2005), feedback training (Carney et al., 2019; Gros et al., 2021; Jowsey et al., 2019; Lee et al., 2021; Meurling et al., 2013; Roberts et al., 2014; Tahtali et al., 2017; Villemure et al., 2019; Wong et al., 2016) and mentoring, and collective meaning-making sessions (Carney et al., 2019).

Among the studies without simulations, studies reported the application of workshops (Christofilos et al., 2015; Marrone, 2018), training sessions with feedback (Braithwaite et al., 2013; Rider et al., 2019; Slater et al., 2012), interactive seminars (Lee et al., 2021; Nagelkerk et al., 2014; Villemure et al., 2019), collaborative activities (Bajnok et al., 2012; Roberts et al., 2014; Slater et al., 2012), and mentoring (Bajnok et al., 2012; Carney et al., 2019). In another study, leadership training and structural changes in the interprofessional context were carried out (Slater et al., 2012). Other researchers conducted case studies (Villemure et al., 2019), collective meaning-search discussions (Carney et al., 2019; Marrone, 2018; Rider et al., 2019), and discussion exercises (Pullon & Fry, 2005).

Regarding the dimensions of the work environment studied, 11 of the papers focused on organizational climate; in all of these papers, there was evidence of a positive effect of IPE, identified in elements such as improved communication, collaboration, and teamwork (Bajnok et al., 2012; Braithwaite et al., 2013; Brewer & Flavell, 2020; Christofilos et al., 2015; Dematteo & Reeves, 2011; Jowsey et al., 2019; Lee et al., 2021; Meurling et al., 2013; Roberts et al., 2014; Tahtali et al., 2017; Villemure et al., 2019; Wong et al., 2016). Additionally, four studies assessing job satisfaction were found (Carney et al., 2019; Marrone, 2018; Meurling et al., 2013; Villemure et al., 2019). Two studies showed positive effects on this dimension after the IPE intervention (Carney et al., 2019; Marrone, 2018), while the other two showed no effect (Meurling et al., 2013; Villemure et al., 2019).

Concerning the studies that analyzed the effect of interprofessional strategies on organizational culture, authors of seven studies found positive effects (Bajnok et al., 2012; Dematteo & Reeves, 2011; Gros et al., 2021; Hinde et al., 2016; Kolbe et al., 2013; Nagelkerk et al., 2014; Rider et al., 2019), and three studies showed mixed results because they found both positive and negative elements that can affect culture (Carney et al., 2019; Jowsey et al., 2019; Slater et al., 2012). The organizational culture components that emerged in our review included cultural change in the organization (Carney et al., 2019; Dematteo & Reeves, 2011; Jowsey et al., 2019), culture aimed at strengthening safety (Hinde et al., 2016; Kolbe et al., 2013; Slater et al., 2012), leaders’ inclusion (Kolbe et al., 2013), roles clarification (Gros et al., 2021) and other non-tangible cultural components of the organization, such as mission (Rider et al., 2019). Lastly, regarding the studies evaluating the effect of IPE strategies on organizational commitment or attachment, two papers found positive effects (Carney et al., 2019; Meurling et al., 2013), and two others explored the positive and negative aspects that could influence organizational attachment, subjectively, both in the present and the future (Christofilos et al., 2015; Pullon & Fry, 2005).

## Discussion

The goal of this review was to gain a better understanding of the effects that IPE can have on several dimensions of health professionals’ work environments. We found 21 qualitative, quantitative, and mixed-method studies of the effects of different IPE strategies on the work environments of health professionals in clinic and hospital settings. The findings evidenced that organization climate and organizational culture were the work environment dimensions showing the most positive effects of IPE (Bajnok et al., 2012; Braithwaite et al., 2013; Brewer & Flavell, 2020; Christofilos et al., 2015; Dematteo & Reeves, 2011; Gros et al., 2021; Hinde et al., 2016; Jowsey et al., 2019; Kolbe et al., 2013; Lee et al., 2021; Meurling et al., 2013; Nagelkerk et al., 2014; Rider et al., 2019; Roberts et al., 2014; Tahtali et al., 2017; Villemure et al., 2019; Wong et al., 2016). For job satisfaction, researchers found both positive (Carney et al., 2019; Marrone, 2018) and no (Meurling et al., 2013; Villemure et al., 2019) effects of IPE. Researchers also found both positive (Carney et al., 2019; Meurling et al., 2013) and no (Christofilos et al., 2015; Pullon & Fry, 2005) effects on organizational attachment.

The mixed results found for job satisfaction and organizational attachment could be due to how these dimensions were measured. For example, neutral results on job satisfaction were found in studies that used quantitative instruments, which may not be sufficient to address the subjectivity of satisfaction in depth (Meurling et al., 2013; Villemure et al., 2019). In contrast, Christofilos et al. (2015), who found mixed results for organizational attachment, used qualitative instruments for data collection and analysis, allowing them to gather subjective opinions about the elements that could facilitate or hinder organizational attachment. In another study, researchers used questions about professionals’ future income and career development to examine organizational attachment; these questions yielded uncertain answers because of the low certainty of future changes (Pullon & Fry, 2005).

Additionally, as shown in Supplementary Material 2, a wide variety of professionals participated in the studies and, following IPE interventions, were able to increase their knowledge about the roles of the other professions (Brewer & Flavell, 2020; Gros et al., 2021; Nagelkerk et al., 2014), learn to work as a team by improving their communication and collaboration skills (Bajnok et al., 2012; Brewer & Flavell, 2020; Gros et al., 2021; Jowsey et al., 2019; Lee et al., 2021; Meurling et al., 2013; Roberts et al., 2014; Slater et al., 2012; Villemure et al., 2019; Wong et al., 2016), and reduce the hierarchies among them (Dematteo & Reeves, 2011).

The included papers addressed specific aspects of each of the work environment dimensions, such as communication and collaborative work in addressing organizational climate (Jowsey et al., 2019; Lee et al., 2021; Roberts et al., 2014; Villemure et al., 2019), or, regarding organizational culture, the culture focused on patient safety (Gros et al., 2021; Wong et al., 2016) and a critique of the culture that emerges when professionals have to work in cubicles (Bajnok et al., 2012). Researchers who addressed organizational attachment examined indicators such as staff turnover, requests for work leave (Meurling et al., 2013), and employee commitment (Christofilos et al., 2015; Pullon & Fry, 2005). More strategies should be created to expand the empirical evidence targeting this dimension, and also job satisfaction, whose results only showed positive effects in some papers and no effect in others. This review used a specific search algorithm because we wished to emphasize the organizational environment results of the four proposed dimensions, and we did not obtain results in the gray literature database that could have broadened the perspective and findings of the review. Therefore, future research should use more sensitive algorithms in order to obtain richer findings.

Although we did identify that IPE programs can have positive effects on the work environments of health professionals, which is in line with the fourth objective of the Quadruple Aim (Interprofessional Educational Collaborative, 2016; Sikka et al., 2015), such strategies require decision-making stakeholders to implement them, both in local and national institutions, as well as in health systems at the global level. Therefore, some authors have also mentioned the importance of decision-makers’ roles in planning, financing, and implementing IPE programs (Bajnok et al., 2012; Christofilos et al., 2015; Dematteo & Reeves, 2011; Wong et al., 2016).

With this review, we will be able to identify IPE strategies that have been shown to have positive effects on the work environments of health care teams, such as simulation (Carney et al., 2019; Dematteo & Reeves, 2011; Gros et al., 2021; Hinde et al., 2016; Jowsey et al., 2019; Kolbe et al., 2013; Lee et al., 2021; Meurling et al., 2013; Nagelkerk et al., 2014; Pullon & Fry, 2005; Roberts et al., 2014; Tahtali et al., 2017; Villemure et al., 2019; Wong et al., 2016) or training sessions with feedback (Braithwaite et al., 2013; Carney et al., 2019; Gros et al., 2021; Jowsey et al., 2019; Lee et al., 2021; Meurling et al., 2013; Rider et al., 2019; Roberts et al., 2014; Tahtali et al., 2017; Villemure et al., 2019; Wong et al., 2016). Knowing which IPE strategies can most effectively improve work environments in health services will help decision-makers meet the third SDG of the 2030 Agenda.

Regarding implications that transcend this study, we highlight that we identified studies from eight different countries, all belonging to the Global North. This review was designed to analyze IPE interventions globally and we included databases that publish in languages other than English, but we still only found research conducted in a small portion of the world. However, in order to comply with the third SDG and, specifically, with Sect. 3.C. (Organización de las Naciones Unidas, 2018), IPE strategies must be implemented globally and not just in high-income countries, whose living and financing conditions are advantageous compared to those of other countries. Future empirical research and reviews could explore the effects of IPE interventions in the work environments of health professionals in the Global South.

In conclusion, IPE training programs have shown mostly positive effects on the organizational climate and culture of clinical and community health care settings. Additionally, IPE has shown some positive effects on organizational commitment and job satisfaction. However, these effects have been studied by heterogeneous methodologies within quantitative, qualitative, and mixed approaches, which enrich the results found. Another interesting finding is that all studies included in this review were run in eight high-income countries, so more research is needed in countries belonging to the Global South, where resources must be well employed. In fact, IPE could be an effective alternative for fulfilling the Quadruple Aim and the third SDG, aimed at improving health and well-being. The IPE needs to be positioned as a trend in global health, aiming at boosting human health resources as one of its Building Blocks and calling the attention of health decision-makers.

### Strengths and limitations of this review

One of the strengths of this review is the inclusion of several databases encompassing the breadth and depth of the literature in different languages (English, Spanish, Portuguese, and French). Including other languages facilitates understanding the different health systems and contextual factors that influence health work environments and IPE outcomes. In addition, identifying IPE strategies that have positive work-environment outcomes for health workers that are in real-world rather than educational settings will, in turn, benefit the patients and health systems.

This review also has several limitations. It is important to note that the methodology used in this study, a scoping review method, may not provide an in-depth analysis of the included studies. Because of the objective of this review, this study offers a broad overview of the existing literature but does not include a critical appraisal of the studies. We found heterogeneity in the included studies regarding their methodologies, settings, and outcome measures, which may affect the comparability and generalizability of the findings. Lastly, this scoping review focused primarily on the effects of IPE on the work environment of health professionals. As it is well-known, studies with negative or inconclusive results may be less likely to be published, leading to a potential publication bias. This publication bias was controlled by our searching grey literature, specifically the Open Gray database. The majority of studies focused on strategies used mostly by physicians and nurses. The latter limits our ability to understand the importance of role recognition within health settings, which is a main objective of IPE. Our scoping review helps us understand the importance of enabling educational strategies with different health professionals and actors contributing to health services in clinical and nonclinical environments. We must also acknowledge that the research team constitutes the interprofessional education team at Universidad del Rosario, which could have influenced our interpretation of the results. Nevertheless, this possible bias was reduced by having one of the investigators (MMC) analyze the data once it was collected in the spreadsheet. She has an education background in psychology and public health and is not part of the interprofessional team at the University. This research was her dissertation for a master’s degree in public health (MMC); therefore, this project was evaluated by two additional independent and anonymous examinators who do not belong to our IPE team. The suggestions made by the reviewers are incorporated into this manuscript.

### Electronic supplementary material

Below is the link to the electronic supplementary material.

Supplementary Material 1

Supplementary Material 2

## Footnotes

1 See https://www.who.int/europe/about-us/our-work/sustainable-development-goals/targets-of-sustainable-development-goal-3.
