# Supplementary material for: The effect of interprofessional education on the work environment of health professionals: a scoping review
Source: Adv Health Sci Educ Theory Pract. 2023 Dec 1;29(4):1463–80. doi: 10.1007/s10459-023-10300-4 (PMC11368981; doi:10.1007/s10459-023-10300-4)
Supplement: Supplementary file 2 — Supplementary Material 2 [file 10459_2023_10300_MOESM2_ESM.docx]

**Online Resource 2. Summary of Results**

| **Dimensions** | **Paper** | **Population** | **Concept: IPE Strategy** | **Context** | **Results** |
| --- | --- | --- | --- | --- | --- |
| **Organizational atmosphere** | Bajnok et al., 2012 | Physicians, nurses, physical therapists, dietitians, recreational therapists PT assistants, audiologists, ultrasonographers, cardiology technologists, clinical laboratory technologists, and social workers. | A mentoring program was carried out to develop and implement the action plan and development goals of the team, as well as the creation of teams and networks. | Regional hospital emergency department, community care center, complex continuing care/rehabilitation center, regional health sciences center, metropolitan hospital wound care team. | Participants agreed with the usefulness of strategy to reach a team agreement. |
|  | Braithwaite et al., 2013 | Physicians, nurses, health and administrative personnel, who constituted the study sample, and support personnel and “others” who did not mention their profession. | 111 IPC initiatives with the working core of collaboration between professionals. | ACT (Australian health service networks). | When comparing the evaluations of the groups of professionals, a significant relationship was found in 9 of 10 cases between the level of belonging and the degree to which they considered that the objectives of the project had been achieved. |
|  | Brewer & Flavell, 2020 | Physiotherapy, nursing, occupational therapy, psychological counseling. | An unspecified IPE strategy was conducted while participants were still students for 10 to 42 days. | Community center, elementary school and senior center. | After the intervention, four factors were found to facilitate interprofessional work: change in attitudes and perceptions, and improved knowledge and skills. Among the attitudinal comments, knowledge of each professional’s role increased, as did confidence and comfort in collaborating with others. |
|  | Christofilos et al., 2015 | Social worker, physical therapist doctor in radiation technology. | Workshops on IPE principles, reflective practice, collaboration, teamwork, IPE facilitation, conflict resolution skills, and feedback. | University Hospital in Toronto. | The results summarized areas for IPE improvement, facilitating factors and hindering factors. |
|  | Dematteo & Reeves, 2011 | Nurses, physicians, social workers, physiotherapists, occupational therapists, pharmacists, psychologists, clinical management professionals, speech therapists, consultants. | Some IPE programs were applied to collaborative leadership development, mentoring between clinicians and junior colleagues, development of clinical environments to promote collaboration, and IPE activities to enhance workplace collaboration. | 13 local university hospitals. | Participants reported improved listening, awareness and inclusion. Additionally, they had a favorable perception of the IPE interventions. However, some participants highlighted concerns about some aspects of the strategy, such as actual application to frontline work and lack of critical analysis of the ‘root’ of organizational change. |
|  | Jowsey et al., 2019 | Management staff, nurses, anesthetists, surgeons, and anesthetic assistants. | A NetworkZ program was carried out with OT personnel,^[[1]](#footnote-1)^ where a simulation was applied to a team, followed by a debriefing. | Operating theaters in 20 District Health Councils. | The summarized results were diverse in terms of organizational atmosphere, showing an improvement in teamwork, communication, workflow, and staff relations after NetworkZ implementation. |
|  | Lee et al., 2021 | Physicians, hospital nurses from each hospital, technician from each hospital. | Stimulation strategy to measure technical knowledge, teamwork collaboration and communication with hands-on training and debriefing. | Non-teaching hospital, Pediatric emergency service. | There was an improvement in teamwork and communication during a 12-month period after the intervention. |
|  | Meurling et al., 2013 | Physicians (specialists and residents in anesthesiology and intensive care), nurses and auxiliary nurses. | On-site simulation-based team training with interactive seminars and simulated scenarios. | ICU^[[2]](#footnote-2)^ at Karolinska University Hospital. | The study found differences in the effect of the intervention between professions. Self-efficacy scores improved among physicians and nurses. The perceived quality of collaboration and communication between nursing assistants and physicians improved. Perception of some environmental factors, such as teamwork atmosphere, safety atmosphere, and working conditions, improved among nursing assistants. The perception of safety by nurses also improved. |
|  | Roberts et al., 2014 | Chief resident, ER technician, GP nurse, junior resident, respiratory therapist. | The training focused on improving leadership, team communication and other characteristics of the role. Training included a simulation followed by a briefing. | The emergency department and simulation center at MMC^[[3]](#footnote-3)^, a teaching hospital affiliated with the Southern Illinois University School of Medicine. | Participants “strongly agreed” with the usefulness of the intervention, except with the statement that the training is a good use of respondents’ time. Participants also agreed with the functionality of the intervention on team functioning. |
|  | Tahtali et al., 2017 | Physicians, emergency room nurses, radiology technicians and medical students. | Stroke team training was conducted with a standardized care protocol, simulation-based training, a theoretical course, and a feedback session. | Hospital (Emergency Department and Stroke Unit). | Most participants rated the intervention as highly positive for their daily practice. |
|  | Villemure et al., 2019 | Beneficiary assistant, respiratory therapist, nurses, anesthesia resident, anesthesiologists. | An intervention based on CRM^[[4]](#footnote-4)^ training was carried out, with an on-site simulation and a subsequent debriefing process. | PACU^[[5]](#footnote-5)^ at the Hospital of the Universities of Montreal and Quebec. | Global IPC and communication showed significant differences between groups. However, the effect of time on communication was not statistically significant, although it did show an improvement. |
|  | Wong et al., 2016 | Nurses and resident physicians. | Pre-strategy development, training sessions, on-site simulations in emergency settings and a reminder strategy with an e-newsletter. | New York Health Sciences Simulation Center (1,200-bed public tertiary care teaching hospital for adults in the United States with an academic affiliation). | T-TAQ:^[[6]](#footnote-6)^ Four of the five teamwork constructs had significant improvement in terms of team structure, leadership, follow-through and mutual support. HSOPS^[[7]](#footnote-7)^: Three of six constructs showed significant improvement. |
| **Organizational culture** | Bajnok et al., 2012 | Physicians, nurses, physical therapists, nursing administrators, occupational therapists, dietitians, recreational therapists, physical therapy assistants, audiologists, utrasonographers, cardiology technologists, medical laboratory technologists, and social workers. | Collective learning and mentoring on the application of team development strategies. | Regional hospital emergency department, community care center, complex continuing care/rehabilitation center, regional health sciences center, metropolitan hospital wound care team. | Participants stated that there were more factors that enhanced the change from “siloed working style” to “teamwork style.” They highlighted positive results related to improved trust, commitment and team pride. |
|  | Carney et al., 2019 | Professors of medicine, nursing, pharmacy and psychology; medical and pharmacy residents; nurses, clinic supervisors, residency program coordinator, medical assistants, and office staff; as well as students in nursing, pharmacy, social work, mental health, and medical assistant programs. | Face-to-face training session, coaching and focus group to evaluate effects. | Primary care residency continuity practice in nine sites representing academic settings and health systems where the PACER project had been conducted. | Cultural factors were analyzed: the culture of change and the culture of learning as facilitating factors for better involvement in IPE strategies. Other cultural factors were perceived as barriers, such as poor communication culture, leadership that did not consider distribution to improve interaction, and lack of facilities for professional interaction. |
|  | Dematteo & Reeves, 2011 | Nurses, doctors, social workers, physiotherapists, occupational therapists, pharmacists, psychologists, clinical management professionals, speech therapists, and consultants. | Debriefing training between three approaches: self-correction, defense inquiry and systemic constructivism. | 13 local university hospitals. | The strategy was seen as a useful tool to reduce hierarchies and improve staff well-being. However, some participants perceived difficulties in applying the intervention on their daily practices and perceived a lack of critical thinking. |
|  | Gros et al., 2021 | Medical doctors (MD), nurses, nursing technicians, anesthesia technicians, operating room assistants, endoscopists, endoscopy technician, technicians, surgical technician student, GI^[[8]](#footnote-8)^, perfusionists, other not specified. | Gastroenterology and general surgery simulations, with three phases (prebrief, simulation and debrief). | Academic medical center, medical setting, laboratory of angiography catheterization (Cath Angio), endoscopy suite, ambulatory surgery center and the main operating room. | Seven categories of improvement were found, including team communication, clarity of the role of professionals and improved preparedness for future reactions. |
|  | Hinde et al., 2016 | Trained nursing staff, health assistants, surgeons and physicians. | On-site simulation in an operating room. | Hospital / operating room. | There was an increase among the security climate media. |
|  | Jowsey et al., 2019 | Management professionals, nurses, anesthetists, surgeons and anesthetic assistants. | A NetworkZ program was conducted with OT staff, in which a simulation was applied to a team, followed by a briefing. | Operating rooms in 20 District Health Councils. | NetworkZ was perceived to have changed the organizational culture by promoting a closer relationship within the teams involved. It was concluded that culture can be both a challenge and an enhancing factor in the success of NetworkZ. |
|  | Kolbe et al., 2013 | Senior anesthesiologists, resident anesthesiologists, anesthesia nurses. | Debriefing strategy with three different cores: self-correction, advocacy, inquiry and, systemic constructivism. | New university hospital, simulation center. | There were significant correlations between leader inclusiveness and the four factors measured. No correlation was found between the measure of psychological safety before and after. However, psychological safety increased significantly from t1 to t2. |
|  | Nagelkerk et al., 2014 | Nursing students, third-year medical students, pediatric residents, and pilot unit staff of registered nurses and nursing technicians. | Staff briefing, video drill and briefing, and a safety round. | Helen DeVos Children's Hospital (HDVCH): 206-bed teaching pediatric hospital. | Participants showed positive perceptions about the usefulness of the strategy to improve factors such as the ability to make decisions, to know information to share with other professionals and technical knowledge. |
|  | Rider et al., 2018 | Physicians, psychosocial staff (social workers, psychologists), nurses, educators, other health professional (medical students, health services researchers) and administrative staff. | A collective construction response was carried out with teamwork and brainstorming, with feedback. | Organization of interprofessional health training (educational). | Five categories were outlined: philosophy and mission, practice and practical strategies, human capital, research productivity and scholarship, and partnership. |
|  | Slater et al., 2012 | Junior physicians, senior physicians, nurses, managers or administrators, pharmacists, occupational therapists, and social workers. | 111 interprofessional collaboration interventions were held. Some of them consisted of panels and meetings between professionals, while others contained feedback sessions. | Healthcare organizations in a city in England, 4 general hospitals, 4 mental health hospitals and 3 sole general practices. | The results showed no changes in the dimensions of culture, such as non-punitive response to error and staff support, among others. However, the paired t-test suggested a significant change in communication. |
| **Work satisfaction** | Carney et al., 2019 | Professors of medicine, nursing, pharmacy, and psychology; medical and pharmacy residents; nurses, clinic supervisors, residency program coordinators, medical assistants, and office staff; as well as students in nursing pharmacy, social work, mental health, and medical assistant programs. | Face-to-face training session. Coaching and discussion group to assess effects. | Primary care residency continuity practice in nine locations representing academic settings and health systems where the PACER project had been conducted. | The results indicated an improvement in job satisfaction, highlighting the effects on team perception that increased the sense of belonging and satisfaction. |
|  | Marrone, 2018 | Doctors, nurses, nursing assistants, executives. | Discussion groups and workshops. | “(…) a large, urban, nonprofit academic medical center in the northeastern United States.” | Satisfaction surveys showed an increase in satisfaction scores among professionals. |
|  | Meurling et al., 2013 | Doctors (specialists and residents in anesthesiology and intensive care), nurses and nursing assistants. | On-site simulation-based team building with interactive seminars and simulated scenarios. | Karolinska University Hospital. | There was no significant effect on SAQ scores. |
|  | Villemure et al., 2019 | Beneficiary assistant, respiratory therapist, nurses, anesthesia resident, anesthesiologists. | An intervention based on CRM training was carried out, with an on-site simulation and a subsequent debriefing process. | PACU at the Hospital of the Universities of Montreal and Quebec. | ISS^[[9]](#footnote-9)^ did not have a significant impact among co-workers. |
| **Organizational commitment** | Carney et al., 2019 | Professors of medicine, nursing, pharmacy and psychology; medical and pharmacy residents; nurses, clinic supervisors, residency program coordinators, medical assistants, and office staff; as well as students in nursing, pharmacy, social work, mental health, and medical assistant programs. | Face-to-face training session. Coaching and discussion group to assess effects. | Primary care residency continuity practice in nine locations representing academic settings and health systems in which the PACER project had taken place. | Openness toward interprofessional intervention was greater among participants with culture of change and culture of learning. |
|  | Christofilos et al., 2015 | Social worker, physiotherapist, nurse, doctor, and doctor of radiation technology. | Workshops on IPW principles, reflective practice, collaboration, teamwork, IPE facilitation, conflict resolution skills, and feedback. | University Hospital in Toronto. | The educational strategy was helpful in identifying areas that might require a commitment to change toward an organizational setting. |
|  | Meurling et al., 2013 | Doctors (specialists and residents in anesthesiology and intensive care), nurses and auxiliary nurses. | On-site simulation-based team training with interactive seminars and simulated scenarios. | Karolinska University Hospital ICU. | Nurse turnover in the intervention group was reduced by an average of 2.2%, compared to an increased percentage in the control group of 1.7%. There was also a significant reduction in sick leave among nurses. |
|  | Pullon & Fry, 2005 | Doctors, nurses, healthcare managers, retirees, paramedics, hygienists and graduate students. | Workshops with theoretical modules on the role of the professions, participation in clinical planning teams and evaluation. Simulated patients were also included. | Primary healthcare. | 40% of respondents stated that they would continue to work in the sector after the intervention. |

1. OT: operating theaters. [↑](#footnote-ref-1)
2. ICU: Intensive care unit [↑](#footnote-ref-2)
3. MMC: memorial medical center [↑](#footnote-ref-3)
4. CRM: Crisis resources management [↑](#footnote-ref-4)
5. PACU: post anesthetic care unit [↑](#footnote-ref-5)
6. T-TAQ: Teamwork Attitudes Questionnaire TeamSTEPPS [↑](#footnote-ref-6)
7. HSOPS: Hospital Survey on Patient Safety. [↑](#footnote-ref-7)
8. Gastroentherologists. [↑](#footnote-ref-8)
9. ISS: Simulación in Situ [↑](#footnote-ref-9)
